# Ecological Risk Assessment of a Metal-Contaminated Area in the Tropics. Tier II: Detailed Assessment

**DOI:** 10.1371/journal.pone.0141772

**Published:** 2015-11-03

**Authors:** Júlia Carina Niemeyer, Matilde Moreira-Santos, Rui Ribeiro, Michiel Rutgers, Marco Antonio Nogueira, Eduardo Mendes da Silva, José Paulo Sousa

**Affiliations:** 1 Centre for Functional Ecology, Department of Life Sciences, University of Coimbra, Coimbra, Portugal; 2 Campus de Curitibanos, Universidade Federal de Santa Catarina, Curitibanos, Santa Catarina, Brazil; 3 National Institute for Public Health and the Environment (RIVM), Bilthoven, The Netherlands; 4 Laboratory for Soil Biotechnology, Embrapa Soybean, Londrina, Paraná, Brazil; 5 Instituto de Biologia, Federal University of Bahia, Campus de Ondina, Salvador, Bahia, Brazil; Chinese Research Academy of Environmental Sciences, CHINA

## Abstract

This study presents data on the detailed evaluation (tier 2) of a site-specific ecological risk assessment (ssERA) in a former smelter area contaminated with metals (Santo Amaro, Bahia, Brazil). Combining information from three lines of evidence (LoE), chemical (ChemLoE), ecotoxicological (EcotoxLoE) and ecological (EcoLoE), in the Triad approach, integrated risk values were calculated to rank sites and confirm the potential risk disclosed with tier 1. Risk values were calculated for the habitat and for the retention functions in each sampling point. Habitat function included the ChemLoE calculated from total metal concentrations. The EcotoxLoE was based on reproduction tests with terrestrial invertebrates (*Folsomia candida*, *Enchytraeus crypticus*, *Eisenia andrei*), shoot length and plant biomass (*Avena sativa*, *Brassica rapa*). For the EcoLoE, ecological parameters (microbial parameters, soil invertebrate community, litter breakdown) were used to derive risk values. Retention function included the ChemLoE, calculated from extractable metal concentrations, and the EcotoxLoE based on eluate tests with aquatic organisms (*Daphnia magna* reproduction and *Pseudokirchneriella subcapitata* growth). Results related to the habitat function indicated that the metal residues are sufficient to cause risk to biota, while the low metal levels in extracts and the general lack of toxicity in aquatic tests indicated a high soil retention capacity in most sampling points. Integrated risk of tier 2 showed the same trend of tier 1, suggesting the need to proceed with remediation actions. The high risk levels were related to direct toxicity to organisms and indirect effects, such as failure in the establishment of vegetation and the consequent loss of habitat quality for microorganisms and soil fauna. This study shed some light on the selection of tools for the tier 2 of an ssERA in tropical metal-contaminated sites, focusing on ecological receptors at risk and using available chemical methods, ecological surveys and ecotoxicity tests.

## Introduction

Ecological risk assessment (ERA) is a process of collecting, organizing and analyzing environmental exposure and effect data to estimate the risk of contamination to ecosystems, being a useful tool, for managing contaminated areas [1]. Only a site-specific ERA (ssERA) integrating contaminant exposure and biological effects, either through toxicity tests or in situ surveys, may reveal potential adverse effects of specific (point or diffuse) pollution problems [2]. Toxicity cannot simply be extrapolated from mixtures of contaminants measured in soil due to interactions between them and potential alterations in their bioavailability caused mainly by soil properties and ageing [3]. Thus, chemical analysis needs to be complemented with ecotoxicity tests, which have the key advantage of assessing the toxicity of the whole soil matrix, including degradation products and metabolites. Moreover, indirect effects of chemicals, like changes in food availability, shifts in species relations and habitat structure, may be more important in ssERA than direct toxicity [4], and such impacts can best be evaluated through *in situ* ecological surveys.

For the process of risk characterization the Triad approach, which consists of integrating three lines of evidence (LoE), chemical (ChemLoE), ecotoxicological (EcotoxLoE) and ecological (EcoLoE) [5], has been highly recommended and successfully applied in ssERA of contaminated soils [1, 6, 7]. The Triad approach is usually applied within a tiered system, i.e., information from each LoE is collected at each tier following a step-wise cost-effective process [1]. While tier 1 is essentially a screening phase, tier 2 is performed to reduce uncertainties about the actual risk. Thus, the tools used in tier 2 to collect information of each LoE should indicate long-term direct or indirect effects of contamination, while being more ecologically relevant and of a high capacity to differentiate levels of contamination [8].

In tier 2, the chemical LoE should comprise extraction techniques to quantify the available fraction of the contaminants in soil, complementing the data obtained with the total contaminant concentrations. This chemical LoE should be complemented with information derived from ecotoxicological tests and ecological surveys. At this phase, the ecotoxicological LoE usually comprises long-term tests focusing on sublethal endpoints to assess both the habitat and retention functions of the soil [8, 9], respectively the ability of soils to serve as habitat for soil organisms and to retain contaminants preventing their mobilization via the water pathway [10].

For the soil matrix, standardized reproduction tests with Oligochaeta [11, 12] and Collembola [13] have been recommended to evaluate sublethal effects on soil fauna (e.g. [14, 15]). Standard tests with plants [16] are also recommended as part of test batteries for the ecotoxicological characterization of soils within ERA processes [17–19], being widely used in toxicity assessments in metal contaminated areas [20–23]. To evaluate the soil retention function, soil extracts are prepared to perform widely established standardized tests with cladocerans and microalgae (e.g., OECD [24, 25]; [26, 27]), as recommended by ISO for the ecotoxicological characterization of soils [10]. Finally, the ecological information collected at tier 2 must provide information on the actual impacts on populations and communities of flora and fauna at the study sites [1]. Surveys of species diversity and community structure of soil invertebrates, soil microbial parameters and decomposition rates are often applied at this LoE. However, when compared with other LoEs, the latter has the disadvantage that is generally very time consuming and may require more specialized knowledge [28].

This study aimed to conduct a tier 2 of a ssERA of a metal-contaminated area in Santo Amaro (BA, Brazil), following the Triad approach, i.e., joining information from the three LoE mentioned above, and complementing the analysis (trying to reduce some uncertainties) done during tier 1 [29]. The results obtained in the screening phase (tier 1) indicated very high risk levels at some sampling points, associated with tailing deposits, which suggested the need to proceed with remediation actions. However, uncertainties generated by contradictory information among the three LoE at certain sampling points indicated the need to further elucidate potential risks through a more detailed assessment (tier 2). This will help ranking sites within the study area and fully identify those that need remediation actions.

## Materials and Methods

### 2.1 Study area

The present study was carried out in an abandoned industrial area in Santo Amaro, BA, Brazil, presenting a severe metal contamination originated from a lead smelter that was operational for 33 years (1960–1993). Human and livestock contamination [30, 31] and very high levels of metals in soil and water [32, 33] have been reported, caused by the tons of contaminated debris deposited around the smelter area (approx. 180,000 m^3^) and under roads and house´s backyards (approx. 55,000 m^3^), as well as by the aerial dispersion and deposition of dusts (covering a larger area up to 3 Km from the area) while the smelter was operational. Soils in the study area are Vertisols and Inceptisols (Soil Taxonomy, USDA) originated from carbonaceous shale, rich in expansive clay (montmorilonite), with generally low porosity and consequently low permeability [34]. More details about the study area can be found in Niemeyer et al. [29].

### 2.2 Soil sampling and selection of reference soils

**Fig 1:**
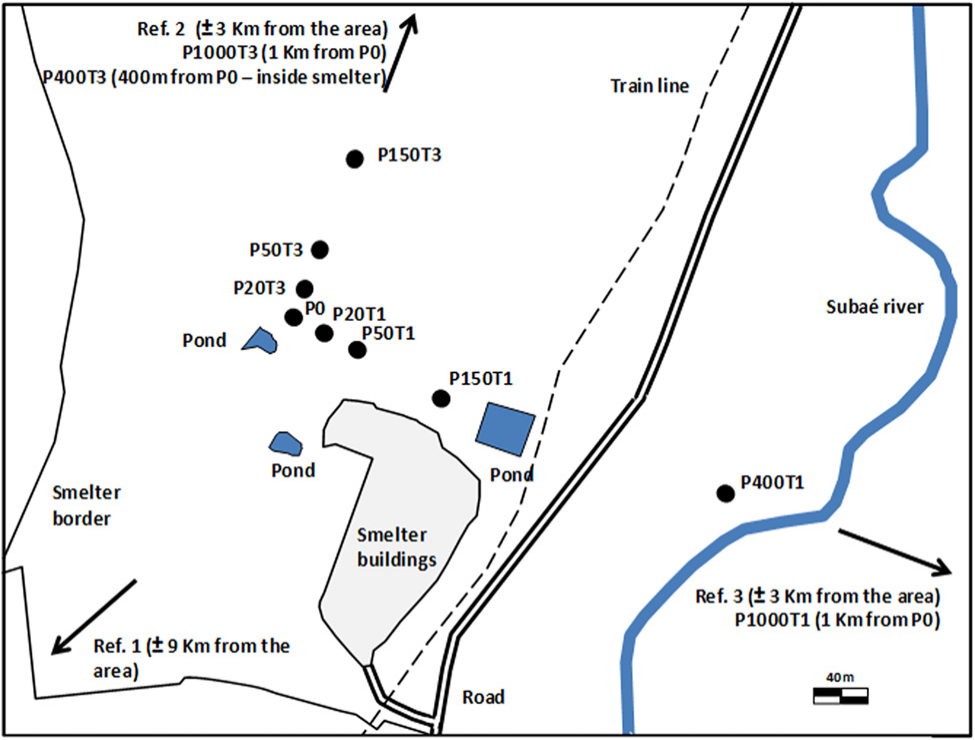
Schematic representation of the study area (an abandoned lead smelter, Santo Amaro, BA, Brazil) showing the location of the 11 sampling points along the two transects and of the three reference points. Font: Julia Niemeyer.

Two 1-km transects (T1 and T3) were defined along the two major detected gradients of contamination (Fig 1). The soil sampling was carried out with court authorization of Brazilian federal justice, 3ª lower court in Salvador, Bahia, Brazil. Field studies did not involve endangered or protected species.

The two transects shared a central point (P0 –located close to the smelter plant) and comprised 5 sampling points, each at 20, 50, 150, 400, and 1000 m from P0. Based on a multivariate factor analysis using soil properties data (metals excluded), soil samples were assembled into three groups mainly differing in terms of texture, organic matter content and pH. Soils from several points in the surrounding of the area were then screened, analyzed for metals and soil properties, and three reference soils (the best possible for each identified group of sampling points) were selected at 3 km (Ref. 2 and 3) and 9 km (Ref. 1) from P0. These were used as “control soils” for each group of soils identified in the factor analysis for the chemical and ecotoxicological lines of evidence, and the average response of the assessed ecological parameters was used as reference values in the ecological line of evidence. Details about soil sampling and soil grouping are shown in Niemeyer et al. [29].

### 2.3 Chemical analysis (ChemLoE)

Based on the historical use of the site and on a previous study [35], soils were analyzed for the four main metals responsible for the contamination of the area (Pb, Cd, Cu, and Zn), and also for Cr, Ni, Fe, Co, and Mn. Metals were quantified in the bulk soil (to evaluate the soil habitat function) and in extracts (to evaluate the soil retention function), obtained by shaking 15 g of soil (dry weight) with 150 ml of a 0.01 M CaCl_2_ solution for 2h:30 min at 200 rpm. The slurry was then centrifuged for 5 min at 3,000 rpm and the extracts (supernatants) were filtered through a Schleicher & Schuell filter paper (Dassel, Germany, Reference n° 595). Metals were quantified in the bulk soil and in extracts by inductively coupled plasma-atomic spectroscopy.

Other soil physico-chemical parameters measured were pH (KCl 1M) [36], water holding capacity (WHC; [37]), cation exchange capacity (CEC; [38]), organic matter (OM) content (loss on ignition at 500°C for 6 h) and soil texture [39].

### 2.4 Soil invertebrate reproduction tests (EcotoxLoE)

Reproduction tests with *Enchytraeus crypticus* (28 d), *Eisenia andrei* (56 d) and *Folsomia candida* (28 d) were conducted to evaluate the soil habitat function following ISO standard guidelines [11–13]. Individuals from these three species were originated from laboratory cultures maintained according the specifications of the corresponding ISO guidelines [11–13]. Tests were conducted at 25°C. Soils were not diluted with reference soil and four or five replicates were prepared with soil from each sampling point (including reference points) and OECD soil (as a positive control). At the end of the exposure period, reproduction was estimated as the number of juveniles per replicate.

Test vessels for *E*. *crypticus* consisted of glass vessels (100 ml capacity) filled with 30 g of soil (wet mass). Ten organisms with a well-developed clitellum were introduced in each vessel and finely ground oat was given as food at the start and 14 d after exposure. The test vessels were opened twice a week to aerate the soil and to adjust its moisture content by weighting the vessels and compensating the weight loss by adding of distilled water. After 28 d, each test container was filled with alcohol 70% to preserve the organisms. Some drops of Bengal red (1% solution in ethanol) were added and the mixture was shaken to homogenize. After 24 h, the content of each vessel was sieved (250 μm) and then the red colored organisms were counted under a stereo microscope (40x). Besides the four replicates prepared with enchytraieds, an additional replicate without organisms was prepared per each test soil to measure soil moisture and pH at the end of the test.

For *E*. *andrei*, one week before starting the reproduction test, adult worms (with a well-developed clitellum) were selected and acclimated in OECD artificial soil (with the addition of fine horse manure as food supply). Four replicates were prepared per test soil, each consisting of a cylindrical plastic box with 500 g of soil (wet mass). At the beginning of the test, ten acclimated worms, weighting between 250 and 500 mg were washed, weighted and then introduced in each replicate. Horse manure was added as food supply once a week. After 28 d, live adult worms were counted. After 56 d, at the end of the assay, the test boxes were placed into a water-bath at 60°C to force juveniles to reach the surface and to be counted.

For the *F*. *candida* reproduction test organisms of 10 to 12 d old obtained from synchronized cultures were used. Five test glass vessels (100 ml capacity) with 30 g of soil (wet mass) were prepared per each test soil. Ten springtails were introduced in each replicate. Granulated dry yeast (approximately 2 mg) was added as food at the beginning and after 14 d of experiment. Twice a week, the test vessels were opened to allow soil aeration and once a week the water loss by evaporation was compensated (water loss determined by the weight loss of the test vessels). After 28 d, the content of each test vessel was transferred to a larger vessel, filled up with water and gently stirred, leading organisms (adults and juveniles) to float into the surface. Afterwards, some drops of a dark ink were added to the water surface to increase contrast and facilitate counting of living organisms. The number of surviving adults was recorded. The water surface was photographed and the number of juveniles was counted using UTHSCSA Image Tool for Windows, version 3.0. As for enchytraeids, an additional replicate without springtails was prepared per each test soil for measuring of soil moisture and pH at the end of the test.

### 2.5 Plant growth tests (EcotoxLoE)

Plant tests (also to evaluate soil habitat function) following the ISO guideline [16] with minor modifications, were used to evaluate the effects on shoot length and biomass of two plant species. The monocotyledonous *Avena sativa* (oat) and the dicotyledonous *Brassica rapa* (rape) were selected, according to a list of species recommended by the ISO guideline. All tests were carried out in undiluted soil samples in plastic boxes (12 cm x 9 cm x 6 cm) filled with approximately 450 g moistened soil (about 50% of the soil WHC), with four replicates per sampling point (including reference points). A number of 10 seeds were planted on each replicate with the help of a pair of tweezers. Each testbox was perforated and a fiberglass rope was inserted into the hole. Then this box was placed inside a similar box filled with distilled water, and the maintenance of soil moisture was guaranteed by capillarity. Twice a week, the position of the test boxes was rearranged according to a randomization scheme, within a plant growth chamber at 23°C with a 16:8-h light:dark cycle (8,000–14,000 lx) and relative humidity of 60%. No fertilizer was added. Seed germination was determined by visual seed emergence. After 50% of the seeds in the control soil had germinated, the number of seedlings per replicate was reduced to 5 evenly distributed plants. After an exposure period of 14 d for *A*. *sativa* and 21 d for *B*. *rapa*, growth was estimated as shoot length (in fresh material) and dry biomass after oven drying the living matter at 70°C until constant weight. OECD artificial soil [40] was used as positive control.

### 2.6 Cladoceran reproduction tests (EcotoxLoE)

The 21-d *Daphnia magna* reproduction tests (soil retention function) were conducted on soil eluates prepared from each tested soil using reconstituted hard water [41], the same used as control and dilution medium in tests; details on eluates preparation are described in Niemeyer et al. [29]. The tests were carried out according to the OECD [28] guideline with 24-h old neonates (clone A*sensu* [42]) from third- to fifth-broods. Ten replicates were set up for each treatment, each with one organism and 50 ml of medium and incubated at 19 to 21°C under a 14:10-h light:dark cycle (4,000 lx). The organisms were fed daily with *Pseudokirchneriella subcapitata* (3 x 10^5^ cells/ml) and newborn neonates were recorded and removed from the vessels. Parent organisms were transferred to new medium every two days, times at which pH, dissolved oxygen and electrical conductivity were measured in the new and old medium. All soil eluates were first tested at 100%. At the end of the 21-d exposure reproduction was estimated as the mean number of offspring per live parent animal. Cases where strong lethal effects were observed (P150T1 and P1000T1), a dilution series of 100, 80, 64, 51, and 40% and 100, 83, 69, 58 and 48% of eluate, respectively, was tested in order to determine the respective median effective dilutions (EC50 values).

### 2.7 Microalgae growth tests (EcotoxLoE)

The 72-h *P*. *subcapitata* (Koršhikov) Hindak growth tests (soil retention function) were conducted on eluates prepared from all soils using distilled water, as described in Niemeyer et al. [29]. The tests were carried out following standard guidelines [24; 43], on 24-well sterile microplates, at 20 to 22°C and under continuous cool-white fluorescent illumination (8,000 lx). Woods Hole MBL growth medium [44] diluted 2.5 times, to keep the required N/P levels, was used as control medium. To exclude the potential confounding effect of differences in nutrient levels across eluates on algae growth, all tests were performed on eluates (only tested at 100%) supplemented with the same amounts of nutrients as in the control medium. Three and six 900-μl replicate cultures were set up per each soil eluate and control, respectively, and each was inoculated with 100 μl of algal inoculum, so that cell concentration at the start of the tests was 10^4^ cells/ml (determined using a Neubauer counting chamber). For further details on testing procedures see Rosa et al. [45]. At the end of the 72-h exposure, algal growth was estimated as the mean specific growth rate per day. Conductivity and pH were measured at the start of each test.

### 2.8 Surface dwelling invertebrates (EcoLoE)

Surface dwelling invertebrates (soil habitat function) were sampled using pitfall traps, which consisted of plastic cups (8 cm diameter and 11 cm depth) filled with alcohol (at 50%) and a few drops of neutral detergent. Three traps were set up at each sampling point, distant from each other by 5 m in a triangular arrangement. After one week exposure, specimens were collected and brought to the laboratory and preserved in alcohol (at 70%) until processing. Collected invertebrates were identified at morphospecies level. For each Order, abundance was estimated as the total number of individuals and richness as the total number of taxa (morphospecies), after summing the results of the three replicates. Details are shown in Niemeyer et al. [46].

### 2.9 Soil microbial parameters (EcoLoE)

The following parameters, involving microbial biomass, enzyme activities and nitrogen transformation rates were determined and used in the risk calculation: microbial biomass of carbon (BMC; μg/g), microbial biomass of nitrogen (BMN; μg/g), asparaginase activity (μg N-NH_4_^+^/g/h), dehydrogenase activity (μg PNP/g/d), acid phosphatase activity (μg PNF/g/h), ammonification rate (μg N/g/d), and nitrification rate (%). Details about soil sampling and determination of soil microbial parameters are outlined in Niemeyer et al. [47].

### 2.10 Organic material decomposition rate (litter mass loss) (EcoLoE)

Litter bags were used to measure litter mass loss (soil habitat function). Nylon bags with a size of 30 cm × 20 cm and a mesh size of 1.0 cm × 0.2 cm were used to allow the decomposition activity both by macro- and microorganisms [48]. Air dried leaves of *Schinus terebinthifolius* Raddi (Anacardiaceae), a native tree species, were collected in a non-contaminated area and used as substrate in the litter bags (4 g in each bag). This species is quite frequent at the study site and is palatable to the soil macrofauna [49]. Litter bags were placed on the soil surface. At each sampling point 4 areas (4 m apart on a quadrangular arrangement) were defined and 4 bags were placed in each area (a total of 16 bags per sampling site). Four litter bags per sampling point (one per area) were collected randomly after exposure periods of 15, 43, 83, and 131 d, and processed immediately. The material was dried at 60°C and weight was recorded. Afterwards, the ash-free dry weight (AFDW) was calculated by subtracting the mass of the ignited residue at 600°C for 1 h. The methodology followed the recommendations of OECD [50] and Römbke et al. [51]. Details about sampling processing and calculations are described in Niemeyer et al. [46]. This parameter was not evaluated in points P20T1 and P20T3 due to the proximity with P0 and P50T1 and P50T3.

### 2.11 Data analysis

#### 2.11.1 Chemical data

Total metal concentrations in soils were compared with HC50_cor_ (Dutch HC50_EC50_ values [52] corrected for sampling site-specific differences, taking into account the organic matter and the clay content of each soil [53]), and the US Ecological Soil Screening Levels (Eco-SSL) for plants [54]. The first were used on risk calculations (see section 2.11.3). The latter were used when analyzing results from plant tests.

#### 2.11.2 Ecotoxicological and ecological data

To avoid repetition with data analysis sections in previous papers of this integrated study the detailed analyses of ecological parameters at each sampling location in comparison with the respective reference (basically using ANOVA, t-tests, or ANOSIM approaches according to the parameters) is outlined in Niemeyer et al. [46] for soil microbial parameters, in Niemeyer et al. [47] for surface dwelling invertebrates and organic material decomposition. Only data analysis for sublethal tests with soil and aquatic organisms and plants are described here. However, even if not described in detail in this paper, all the ecological parameters presented in this materials and methods section were used to derive risk values for the ecological LoE and the integrated risk values (see section 2.11.3).

For the ecotoxicological tests (invertebrate reproduction, plant growth, cladoceran reproduction and microalgae growth), differences among contaminated soils and the respective reference soil were evaluated by one-way analysis of variance (ANOVA), followed by one-tailed Dunnet’s test when necessary. Organic matter content was used as covariable when analyzing plant biomass and shoot length. Prior to all analysis, normality and homoscedasticity were checked via the Shapiro-Wilk’s test and Bartlet test, respectively. When homoscedasticity was not fulfilled, an equivalent non-parametric tests was used, namely the Kruskal-Wallis ANOVA followed by Dunn’s multiple comparisons test.

For the cladoceran reproduction tests on a range of eluate dilutions the median and 20% effective dilutions (EC50 and EC20, respectively) and respective 95% confidence limits (CL) were obtained by fitting organism responses to a logistic model using the least squares method [25]. All statistical analyses were carried out using the Statistica 7.0 software (Staf Soft).

#### 2.11.3 Risk calculations

All parameters measured for each LoE were used for risk calculations where risk values are expressed in a scale ranging from zero (“no risk”) to one (“highest risk”) [1]. This method assumes that the risk value of reference soils is zero, thus the risk of test soils is always given in relation to the value of the respective reference soil. It implies that all results from the different tests should be made comparable (expressed on the same scale) across the various LoE.

For each sampling point, risk values were calculated following three steps: (1) scale the results (between 0 and 1) of each test/parameter within each LoE; (2) integrate all scaled information of all parameters within each LoE to calculate the risk derived for each LoE; (3) integrate the information from the three LoEs and calculate the integrated risk. In the present study, the integrated risks to the soil habitat and retention function were calculated separately.

In the first step, the results of all tests/evaluation within each LoE were scaled between 0 and 1. For the ChemLoE of the habitat function, the total concentrationof each metal was used to calculate the specific Toxic Pressure (PAF—Potential Affected Fraction of species) at each sampling point, in the same way as done in tier 1 [29]. Benchmarks (HC50_cor_ values, i.e., HC50_EC50_ corrected for organic matter and clay contents of each soil from each sampling point) and model parameters used for each metal in these calculations can be found in Rutgers et al. [52]. For the ChemLoE of the retention function, results from each extractable metal were compared to water quality objectives extracted from VROM [55] and then scaled against metal values from eluates from the respective reference soils according to Jensen and Mesman [1].

For the EcotoxLoE of both the habitat and retention functions, results of the ecotoxicological tests were used and scaled between 0 and 1. Negative values (increase relatively to reference) were set to zero. For habitat function, absolute data on reproduction of *E*. *andrei*, *E*. *crypticus* and *F*. *candida*, and on growth, both as shoot length and biomass, of *A*. *sativa* and *B*. *rapa* were scaled. For the retention function, the effects of the eluates on *D*. *magna* reproduction and *P*. *subcapitata* growth, expressed as the percentage of inhibition in comparison to the control, were used.

For the EcoLoE, only the risk to the habitat function was calculated, by scaling the data on surface dwelling invertebrates, soil microbial parameters and organic matter decomposition relatively to the overall reference value. Data on abundance and morphospecies richness of the most frequently soil surface dwelling groups (Araneae, Hymenoptera, Coleoptera, Orthoptera) were used separately, while data on other groups, including Isopoda, Dermaptera, Hemiptera, Diplopoda and Mantodea, were pooled. Since both abundance and number of morphospecies are the result of the same survey, the BKX_Triad method [1] was used. This method allows integrating information from different ecological observations into a single risk value, even if the original data has different units.

In the second step, the risk for each LoE was calculated by integrating the respective scaled information for each parameter [1]. In the ChemLoE for the habitat function this was achieved by integrating the individual metal risk according to a response addition model [56].

Finally, in step three, the integrated risk (IR) for habitat and retention functions were calculated for each tested soil (sampling point) independently and using the risk values from each LoE (ChemLoE, EcotoxLoE and EcoLoE in the case of habitat function, but only ChemLoE and EcotoxLoE in the case of retention function). To evaluate whether the different LoEs contributed differently to the total risk, the standard deviation associated to each IR value was also calculated. More details on the calculation involved in each of the three steps (including formulas for each type of data used) can be seen in the **S1 File.**

## Results and Discussion

### 3.1 Sites characterization and metal concentrations (ChemLoE)

Overall, soils from the study sites showed high clay percentage (above 30%, except for group 2 soils with values close to 10%) low (<2%) to medium (2 to 6%) organic matter content [54], a cation exchange capacity (CEC) mostly between 30 and 40 meq/100 g, and pH values near to neutral, with the exception of sites P1000T1 and Ref. 2 with a low pH of 3.7 and 4.9, respectively (Table 1). These characteristics agree with those reported by Anjos [32], who identified basic pH, high CEC, high clay percentage, and high organic matter content as characteristics of soils from the study area.

**Table 1:** Physico-chemical characteristics of the three groups of soils sampled at the Santo Amaro (BA, Brazil) study area and respective reference soils. USDA–United States Department of Agriculture; CEC–Cation Exchange Capacity; WHC–Water Holding Capacity.

Soil group	Coarse sand (%)	Fine sand (%)	Sand (total) (%)	Silt (%)	Clay (%)	Texture (USDA)	CEC (meq 100g)	pH (KCl 1:5 v:v)	P (mg/kg)	Organic matter (%)	Mineral N (mg/kg)	Water content (%) (1)	WHC (g/100g)
**Group 1**													
Ref 1	2.3	8.5	10.9	42.1	47.0	Silty Clay	34.16	7.1	72	1.1	42	19.54	53.78
P1000T1	2.5	21.8	24.3	19.9	55.8	Clay	43.20	3.7	35	2.0	56	28.74	59.95
P20T3	11.4	30,0	41.4	22.3	36.3	Clay Loam	42.16	6.8	106	1.9	42	35.04	67.73
P400T3	6.5	8.6	15.1	52.4	32.5	Silt Clay Loam	35.84	7.1	1	1.9	70	45.48	56.67
**Group 2**													
Ref 2	50.9	38.5	89.4	2.8	7.7	Loamy Sand	37.60	4.9	1	1.0	42	13.21	27.53
P0	43.2	31.3	74.5	11.9	13.6	Sandy Loam	38.56	6.7	47	0.3	70	31.04	44.12
P20T1	48.0	13.8	61.8	19.0	19.3	Sandy Loam	37.28	7.1	58	0.2	42	32.67	46.40
P150T1	56.2	21.1	77.4	12.3	10.3	Sandy Loam	21.28	6.7	>200	2.1	42	29.41	28.55
P50T3	69.2	9.1	78.3	10.4	11.3	Sandy Loam	16.56	7.2	>200	2.8	56	39.48	22.05
**Group 3**													
Ref 3	22.2	15.0	37.2	11.1	51.7	Clay	36.48	6.1	52	3.9	56	47.20	60.75
P50T1	25.2	13.4	38.6	29.0	32.4	Clay Loam	38.16	6.7	63	1.1	56	28.59	54.51
P400T1	19.6	23.9	43.5	20.2	36.3	Clay Loam	37.44	6.8	>200	5.1	56	24.43	58.93
P150T3	8.4	15.2	23.5	21.4	55.1	Clay	49.20	6.8	16	2.5	42	40.71	61.76
P1000T3	10.3	19.5	29.8	29.8	40.4	Clay Loam	42.72	7.0	>200	5.7	42	n.d.	57.57

(1) Soil moisture in the samples used for microbial assessments.

Total and extractable metal concentrations are shown in Table 2. For at least one among four metals (Pb, Cd, Cu, and Zn), soils from three sampling points, within group 2 presented levels exceeding by far the benchmark HC50_cor_ values. However, low metal concentrations, the vast majority below detection levels (the few exceptions were found at P1000T1, P150T1, P50T3, Ref 3, and P150T3), were found in 0.01 M CaCl_2_ extracts (Table 2), indicating a probable high metal adsorption to soil particles, which is in accordance to the type of expansive clay (montmorillonite) of high plasticity found in this region [34], which probably has increased by ageing (since the smelter ended its activities in 1993).

**Table 2:** Total and extractable metal concentrations in the three groups of soils sampled at the Santo Amaro (BA, Brazil) study area and respective reference soils.

Sites	Total (mg/kg)								Extractable (mg/l) in 0.01 M CaCl_2_ (1:10; v:v)							
	Pb^a^	Cd^a^	Cu^a^	Zn^a^	Cr	Ni	Fe	Mn	Pb	Cd	Cu	Zn	Cr	Ni	Fe	Mn
**Group 1**																
Ref 1	16	<0.2	66	94	77	54^b^	45000	840^b^	<0.1	<0.01	<0.8	<0.2	<0.8	<1.4	<1.1	<0.5
P1000T1	23	<0.2	60	80	62	46^b^	48000	360^b^	0.1	0.4	<0.8	1.9	<0.8	1.6	<1.1	71
P20T3	308^b^	<0.2	56	420^b^	78	60^b^	49000	672^b^	<0.1	<0.01	<0.8	<0.2	<0.8	<1.4	<1.1	<0.5
P400T3	179^b^	0.3	44	90	59	46^b^	34000	760^b^	<0.1	<0.01	<0.8	<0.2	<0.8	<1.4	<1.1	<0.5
**Group 2**																
Ref 2	13	<0.2	18	24	16	28	2900	34	<0.1	<0.01	<0.8	0.2	<0.8	<1.4	<1.1	0.8
P0	1264^b^	<0.2	76^b^	3800 ^(2.8)^ ^b^	72	57^b^	52000	674^b^	<0.1	<0.01	<0.8	<0.2	<0.8	<1.4	<1.1	<0.5
P20T1	133^b^	<0.2	56	220^b^	80	56^b^	41000	780^b^	<0.1	<0.01	<0.8	<0.2	<0.8	<1.4	<1.1	<0.5
P150T1	37460 ^(10.4)^ ^b^	771 ^(9.8)^ ^b^	594 ^(1.6)^ ^b^	42200 ^(33.5)^ ^b^	57	70^b^	110000	1720^b^	2.2	7.3	<0.8	1.3	<0.8	<1.4	<1.1	<0.5
P50T3	26074 ^(7.1)^ ^b^	62^b^	3196 ^(8.2)^ ^b^	95940 ^(73.5)^ ^b^	80	40^b^	117000	5880^b^	<0.1	<0.01	<0.8	1.0	<0.8	<1.4	<1.1	<0.5
**Group 3**																
Ref 3	152^b^	<0.2	40	260^b^	59	40^b^	53000	820^b^	<0.1	0.28	<0.8	<0.2	<0.8	<1.4	<1.1	1.3
P50T1	164^b^	<0.2	60	240^b^	80	58^b^	43000	720^b^	<0.1	<0.01	<0.8	<0.2	<0.8	<1.4	<1.1	<0.5
P400T1	961^b^	8.8	60	840^b^	64	48^b^	35000	540^b^	<0.1	<0.01	<0.8	<0.2	<0.8	<1.4	<1.1	<0.5
P150T3	2200^b^	12	108^b^	3300^b^	84	58^b^	56000	678^b^	<0.1	<0.01	<0.8	0.2	<0.8	<1.4	<1.1	<0.5
P1000T3	99	<0.2	56	156	84	52^b^	49000	568^b^	<0.1	<0.01	<0.8	<0.2	<0.8	<1.4	<1.1	0.9

^a^Numbers in superscript indicate the exceedance (multiplication factor) relatively to the HC50_cor_ (corrected Dutch HC50_EC50_ values after Rutgers et al. 2008) (Ex: the [Pb] at P150T1: 37460 ^(10.4)^, indicates that [Pb] was 10.4 times higher than the HC50_cor_ for Pb).

^b^Total metal concentrations in several sampling points exceeded the Eco-SSL for plants proposed by USEPA (2004): Pb: 120 mg/kg soil; Mn: 220 mg/kg soil; Cd: 32 mg/kg soil; Cu: 70 mg/kg soil; Ni: 38 mg/kg soil; Zn: 160 mg/kg soil.

### 3.2 Soil invertebrate reproduction tests (EcotoxLoE)

The validity criteria as defined in the ISO guidelines for the tests with the three soil invertebrates were met both on OECD and reference soils. Results on the reproduction of *E*. *crypticus*, *E*. *andrei* and *F*. *candida* are shown in Table 3.

**Table 3:** Number of juveniles of soil invertebrates, and plant shoot length and biomass (average ± standard deviation) in ecotoxicity tests for the assessed sampling points. Asterisks indicate significant differences (* p<0.05; ** p<0.01; *** p<0.001) for a one-tailed hypothesis of a Dunnet’s test between each sampling point and the respective reference soil. In the ANOVA for *E*. *andrei* and for plants, soil organic matter was used as covariable. n—number of replicates.

	Reproduction tests (mean number of juveniles)			Shoot length of plants (cm)		Biomass of plants (g dry weight)	
Soil groups	*E*. *crypticus* (n = 4)	*E*. *andrei* (n = 5)	*F*. *candida* (n = 5)	*A*. *sativa* (n = 4)	*B*. *rapa* (n = 4)	*A*. *sativa* (n = 4)	*B*. *rapa* (n = 4)
**Ref 1**	583.0 ± 121.1	70.3 ± 9.5	662 ± 161.3	31.7 ± 1.8	3.3 ± 0.2	0.17 ± 0.01	0.17 ± 0.04
P1000T1	121.8 ±34.9**	5.3 ± 7.1***	642 ± 91.9	39.5 ± 5.3	2.7 ± 0.2**	0.31 ± 0.04	0.07 ± 0.02***
P20T3	896.0 ± 263.5	53.8 ± 12.5	408 ±185.9*	18.4 ± 1.0***	2.7 ± 0.2**	0.11 ± 0.01**	0.07 ± 0.01***
P400T3	599.3 ± 180.0	71.0 ± 17.5	377 ±89.3**	23.0 ± 1.9**	2.3 ± 0.3***	0.16 ± 0.01	0.06 ± 0.01***
**Ref 2**	1089.3 ± 86.4	132.3 ± 24.7	224 ±61.8	25.2 ± 3.9	2.7 ±0.2	0.17 ± 0.02	0.09 ±± 0.02
P0	536.5 ± 144.6***	91.0 ± 15.4**	760 ±124.1	26.3 ± 2.1	3.7 ±0.4	0.23 ± 0.02	0.24 ± 0.02
P20T1	452.0± 36.0***	97.3 ± 20.5*	494 ±105.1	25.2 ± 2.3	2.9 ± 0.0	0.20 ± 0.03	0.14 ± 0.01
P150T1	7.3 ±0.5***	10.3 ± 1.7***	613 ±55.4	19.8 ± 1.9*	2.7 ± 0.2	0.14 ± 0.04	0.04 ± 0.01**
P50T3	450.8 ± 64.6***	45.5 ± 8.3***	411 ± 135.8	19.5 ± 3.3*	2.9 ± 0.2	0.17 ± 0.01	0.06 ± 0.01
**Ref 3**	854.7 ± 421.3	122.5 ± 25.0	890 ± 103.2	34.8 ± 3.7	4.7 ±1.2	0.26 ± 0.05	0.45 ± 0.17
P50T1	560.0 ± 164.3	97.0 ± 11.2*	351 ± 141.5***	25.3 ± 2.9**	4.3 ±0.2	0.17 ± 0.03*	0.32 ± 0.05
P400T1	773.5 ± 175.4	103.0 ± 12.8	831 ± 87.9	42.7 ± 3.0	7.3 ± 0.4	0.29 ± 0.05	0.91 ± 0.08
P150T3	615.8 ± 196.1	20.3 ± 5.7***	344 ± 42.9***	28.9 ± 1.0*	3.1 ± 0.1**	0.16 ± 0.03*	0.16 ± 0.02***
P1000T3	555.0 ± 34.5	85.3 ± 4.6**	577 ±121.9***	30.9 ± 5.3	4.8 ± 0.2	0.24 ± 0.05	0.30 ± 0.04*

*E*. *crypticus* showed a significantly lower reproduction in soils P1000T1, P0, P20T1, P150T1, and P50T3 (all belonging to the second group, except P1000T1) when compared to the respective natural reference soil. The highest mean number of juveniles/replicate was found in Ref 2, 1089 (±86), while the most toxic soil was P150T1, with just 7 (±0.5) juveniles/replicate. Significant lower reproduction of *E*. *andrei* was observed in the same soils plus in soil P50T1, P150T3 and P1000T3 (group 3). The highest mean (± SD) number of juveniles/replicate was found also in Ref 2, 132 (±25), while the most toxic soil was P1000T1, where just 5 juveniles/replicate were observed.

The inhibition of the reproduction of both oligochaete species in soils P0, P150T1 and P50T3 was expected, as these were the most metal contaminated soils, exceeding the benchmark HC50_cor_ values. However, the impairment of reproduction in soil P1000T1, not contaminated by metals, could most likely be related to properties of the soil acting as limiting factors for these species, namely low pH (3.7) combined with low OM (2.0%) and high clay (55.8%) contents. The limitations on the use of *E*. *andrei* in strongly acid soils or soils with low organic matter content has been previously reported [57–59]. Although *E*. *crypticus* presents a broader tolerance than *E*. *andrei* to different soil properties (e.g. range of 4.2–7.7 for pH, 0.6–4.8% for OM, and 3–49% for clay) [59], the characteristics of P1000T1 soil were out of its range of tolerance. Moreover, the effects observed in P1000T1 could be also related to the presence of contaminants not analyzed, namely pesticides, since this point is located in a pasture area [29]. The effects on the reproduction of both oligochaete species observed in P20T1 soil could be related to its low OM content (0.2%).

A different trend was observed for the reproduction of *F*. *candida*. A significant lower reproduction was observed in soils P20T3, P400T3 (both from Group 1), P50T1, P150T3, and P1000T3 (all from Group 3), when compared to the respective natural reference soil. The highest and lowest mean (±SD) number of juveniles/replicate was found in Ref 3 and Ref 2, 890 (±103) and 224 (±62), respectively. The rather low reproduction in Ref 2 was probably one of the reasons for the existence of non significant effects in this group of soils since, in effect, in these soils at least one metal (Pb, Cd, Cu, or Zn) exceeded in its concentration the HC50_COR_ values.

In general, *F*. *candida* appeared to be less sensitive to metal contamination than *E*. *andrei* and *E*. *crypticus*. Similar results were also found by Schultz et al. [60], reporting *Enchytraeus* sp. to be more sensitive than *F*. *candida* in metal-contaminated soils. Also, Van Gestel et al. [17] observed that Collembola appeared to be less sensitive than earthworms and plants to assess soils toxicity with oil and metal contamination. Differences in the sensitivity of collembolans and oligochaetes on metal contaminated soils could be explained in part by differences in exposure [18], since soil solid phases are more important for the uptake process of collembolans, while soft-body oligochaete species are more influenced by porewater characteristics [61]. It is also suggested that Collembola can avoid contaminated food, and are able to excrete assimilated metals at moulting [62], which subsequently can be related to their low sensitivity to metal contamination. A decline of the reproduction of these organisms in metal contaminated sites suggests impacts on habitat function for these groups, which can affect soil functions related to decomposition of organic matter, nutrient cycling and soil aeration.

### 3.3 Plant growth tests (EcotoxLoE)

The growth results, both as shoot length and biomass, of *A*. *sativa* and *B*. *rapa* in all tested soils and respective references are shown in Table 3. Species and endpoints were affected differently, though generally soils from the first group were found to be more toxic. Significant lower plant growth was observed in the soils P1000T1, P20T3 and P400T3 (from the first soilgroup), soils P150T1 and P50T3 (from the second soil group) and soils P50T1, P150T3 and P1000T3 (from the third soil group). These results were expected since total concentrations for some of the metals in all these soils exceeded several metal Eco-SSL for plants (Table 2). However, not all soils with exceedance of these benchmarks demonstrated reduced plant growth (P0, P20T1 and P400T1). These results highlight the fact that exceedance of Eco-SSL does not necessarily mean risk, most likely due to modifications in the bioavailability of metals by the soil properties and/or to the complex effect of mixtures of contaminants [9].

The results of the present study also show that the effects of metal contamination were species-specific. This finding is in agreement with the study of An [63] who investigated the toxicity of Pb and Cu to four plant species (*Sorghum bicolor*, *Cucumis sativus*, *Triticuma estivum*, and *Zea* mays) and found that Pb and Cu showed either antagonistic or synergistic toxic effects depending on the plant species. Also Ben Ghnaya et al. [64] stated detrimental effects of the metals Zn and Cd on the growth, chlorophyll and carotenoid content and metal accumulation on four varieties of *Brassica napus* depending on the metal and plant variety.

Nevertheless, it should be stressed that toxic effect on plants can be related not only to metal contamination, but also to other soil factors like a lack of soil nutrients and/or modified soil physical properties. The latter are also common problems in mined areas with tailing deposits. For instance, Gong et al. [65] evaluated four plant species in 15 soils, including five mineral oil-contaminated soils, and concluded that soil nutrient status rather than soil texture significantly affected both seedling emergence and shoot biomass. Also, results obtained by Alvarenga et al. [23] showed that negative effects on the growth of *Lepidium sativum* in mine soils were probably due not only to metals and soil acidity, but also to the lack of porosity and proper soil structure. Thus, in the present study, the low organic matter content and low WHC at most sampling points combined with metal levels, could be responsible for the observed detrimental effects on plant growth.

### 3.4 Cladoceran reproduction and microalgae growth tests (EcotoxLoE)

All tests conducted with the eluates from all the soils fulfilled the validity criteria established in the guidelines for cladoceran and microalgae control performance. Significant effects on the reproduction of *D*. *magna* were found with eluates from soils P1000T1 (with 100% mortality at the 100% dilution) and P150T1 (with 30% mortality and 41% inhibition on reproduction at the 100% dilution). In all other tested dilutions of P1000T1 and P150T1 eluates and 100% dilution of all the remaining soil eluates mortality was below the control validity criterion of 20% and no significant effects on reproduction were found. As a result, EC20 and EC50 values for P1000T1 eluate were much higher than the 100% dilution (149 and 852%, without a significant regression) and for P150T1 eluate were 88% (95%CL: 60–115) and also higher than 100%, respectively (both without a significant regression). The lethal toxicity here observed with these eluates was lower than that found for the lethal tests of tier 1 with 48 h LC50 (median lethal concentration) values of 91 and 68% for P1000T1 and P150T1 eluates, respectively [29]. This fact can be explained by the adsorption of metals to the surface of microalgae cells (added daily as food) in the reproduction test, turning them less bioavailable, while in the lethal test no food was provided during exposure. In a study on the influence of algal biomass on metal adsorption [66], the green alga *Chlorella minutissima* to rapidly adsorb more than 90% of the initial Pb concentration in solution, which reached the equilibrium within minutes. Kaulbach et al. [67] studying the adsorption of Cd onto the cell wall of *P*. *subcapitata*, showed the importance of microalgae in controlling the transport and fate of metals in the environment.

In the *P*. *subcapitata* growth test increases in cell density by at least a 40-fold factor with coefficients of variation of the mean specific growth rate lower than 4% (performance above that required for control growth), were observed in all tested eluates, suggesting the absence of toxicity. However, significant inhibitions in growth (higher than a 10% threshold considered as ecologically acceptable) were observed in P1000T1 and P150T1 eluates, with 16 and 20% inhibition relatively to the respective reference, respectively. These results corroborate those of the lethal (in tier 1; [29]) and of the present (tier 2) reproduction tests with *D*. *magna*, strongly indicating toxicity in both eluates which exceeded the water quality objectives [55]. However, as already indicated before [29], the response observed in P1000T1 eluate could also be related either to the low soil pH (3.7) or to other not analyzed contaminants (this sampling point was located in a pasture area; Tables 1 and 2). Overall, the present results suggest that the retention function of soils at most sampling points prevented the mobilization of metals via the water pathway, especially to groundwater, a finding in agreement with the results of the ChemLoE reporting low amounts of extractable metals.

### 3.5 Surface dwelling invertebrates (EcoLoE)

A total of 1,277 individuals, separated into 72 morphospecies of soil invertebrates were collected in the pitfall traps. Hymenoptera, Coleoptera and Orthoptera were found at all sampling sites, Araneae at 92% of them, while the group pooling Isopoda, Dermaptera, Hemiptera, Diplopoda, and Mantodea was also found at all sampling points (Table 4; for more detailed results please see Niemeyer et al. [47]).

**Table 4:** Total number of individuals and morphospecies (shown in brackets) of surface dwelling invertebrates caught in pitfall traps (n = 3) at each 11 of the 13 sampling point. Main Orders are presented individually while less abundant orders (Isopoda, Dermaptera, Hemiptera, Diplopoda,Mantodea and Opilionidae) were pooled and presented in a single group called Others. The values for the reference points were averaged to give an overall reference value. No data was obtained for Ref 3 sampling point due to the loss of all pitfall traps due to animal trampling.

	Ref 1	Ref 2	Overall reference	P0	P20T1	P20T3	P50T1	P50T3	P150T1	P150T3	P400T1	P400T3	P1000T1	P1000T3
Orders														
Araneae (Ar)	73 (5)	9 (4)	41 (5)	4 (3)	2 (2)	7 (4)	2 (1)	3 (1)	5 (4)	0 (0)	14 (4)	5 (5)	2 (2)	139 (7)
Hymenoptera (Hy)	15 (5)	15 (5)	15 (5)	31 (3)	26 (3)	48 (4)	76 (3)	57 (5)	33 (2)	52 (4)	15 (3)	16 (6)	45 (5)	30 (3)
Coleoptera (Co)	11 (4)	13 (6)	12 (5)	8 (4)	2 (2)	28 (7)	16 (5)	32 (7)	2 (1)	14 (5)	88 (4)	13 (3)	7 (4)	31 (4)
Orthoptera (Ort)	14 (3)	10 (3)	12 (3)	16 (4)	8 (3)	21 (3)	13 (2)	21 (6)	4 (2)	9 (2)	10 (2)	16 (2)	24 (2)	23 (4)
Others	3 (1)	8 (5)	6 (3)	1 (1)	3 (3)	11 (3)	6 (2)	4 (1)	1 (1)	7 (2)	14 (5)	23 (5)	15 (4)	3 (3)

Araneae presented the highest abundance (139 individuals) and number of taxa (7 morphospecies) in P1000T3 soil, but its abundance and richness was generally low at all other sampling points and no organisms from this Order were found in P150T3 soil. For both Coleoptera and Orthoptera as well as for the “Others”, the lowest abundance and richness were found in P150T1 soil (2 and 1, 4 and 2, and 1 and 1, respectively). On the contrary, Hymenoptera presented the highest abundance (76 individuals) inside the smelter area, at point P50T1, though the highest number of taxa (6) was registered at point P400T3. The lowest abundance and richness within this Order was found at point P400T1. For the latter three groups the highest invertebrate abundance and richness were found either in transect T1 but far from the smelter area or in transect T3. Overall, higher abundance of Araneae, Coleoptera and Orthoptera was observed outside rather than inside the smelter area, especially along transect T1.

Different guilds responded differently to contamination and the ground-hunting organisms were the most affected. The decrease in abundance (241 individuals outside vs. 23 inside) and species richness (16 outside vs. 8 inside) inside the smelter area can be attributed to both direct and indirect effects. Depletion of preys for specialist species, and the impoverishment of the habitat structure, may have impaired the trophic and habitat requirements for many species. The inverse trend observed for abundance of ants is in agreement with Grzes [68], who found an increase in species richness along a metal contamination gradient. An explanation could be that ants have the ability to regulate metals and resist in metal contaminated sites [68, 69]. Furthermore, indirect effects on ant population may have occurred, since ants may have benefited from the decrease in abundance or richness of spiders and coleopterans, as these groups are known to be predators or competitors of ants. In addition, metal pollution affected the habitat structure, creating patches of low vegetation cover, resulting in increase in soil temperature and decrease in moisture content, which may have favored thermophilic species that may exist in the area [68]. Additional analysis and more details are shown in Niemeyer et al. [47].

### 3.6 Soil microbial parameters (EcoLoE)

Microbial community was highly impaired by metal contamination, since all microbial parameters inside the smelter area were significantly affected relatively to the overall reference value, whereas outside the smelter area such effects were rarely observed (Table 5). The significant negative correlations between all except one of the microbial parameters and the metal concentrationsreported by Niemeyer et al. [46] illustrated the detrimental effects of metal contamination on the soil microbial community, and, subsequently on the biogeochemical cycles. Therefore, in general the present results of tier 2 corroborate those found in tier 1, where the soil basal respiration rate was lowest in the metal contaminated soils inside the smelter area [29] and correlated negatively with total soil metal concentrations [46].

**Table 5:** Soil microbial parameters and organic material decomposition (mean ± standard deviation) for the assessed sampling points. The values for the three reference points were geometrically averaged to give an overall reference value. Asterisks indicate significant differences (* p<0.05; ** p<0.01; *** p<0.001) for a one-tailed hypothesis of a Dunnet’s test between each sampling point and the overall reference value (assuming that Ref value higher than sampling point value and lower for Potential Nitrification). In the ANOVA for soil microbial parameters, soil moisture, soil organic carbon and soil nitrogen contents were used as covariables (data extracted from Niemeyer et al 2012a,b). n—number of replicates.

Soil groups	MBC (μg/g) (n = 3)	MBN (μg/g) (n = 3)	Asparaginase (μg N-NH_4_^+^/g/h) (n = 3)	Dehydrogenase (μg PNP/ g/ d) (n = 3)	Acid phosphatase (ug PNF/g/h) (n = 3)	Ammonification (ug N g^-1^ day-^1^) (n = 3)	Nitrification (%) (n = 3)	Decomposition rate ^a^ *k* (monthly) (n = 4)
**Overall reference**	642.4 ± 416.1	50.1 ± 16.2	84.9 ± 53.2	7.2 ± 2.3	617.1 ± 233.2	0.7 ± 0.3	3.9 ± 2.2	0.2656 ± 0.1438
P0	178.1 ± 55.1***	5.4 ± 2.8***	53.8 ± 29.5	0.7 ± 0.4 **	269.0 ± 22.1**	1.7 ± 0.2	15.2 ± 9.6**	0.04667***
P20T1	252.4 ± 142.3**	12.8 ± 4.6***	15.9 ± 18.4***	1.3 ± 1.9**	196.4 ± 33.9***	1.1 ± 0.5	12.4 ± 2.1*	n.d.
P20T3	170.3 ± 174.1 ***	18.6 ± 3.5***	71.0 ± 12.7	1.4 ± 1.0**	443.4 ± 9.3	1.5 ± 0.3	13.3 ± 3.5*	n.d.
P50T1	412.9 ± 31.4	11.0 ± 4.2***	11.2 ± 19.5***	1.2 ± 2.0**	235.7 ± 50.3**	1.8 ± 0.3	17.1 ± 4.9***	0.0632***
P50T3	461.7 ± 20.1	22.0 ± 4.5*	32.5 ± 35.1**	2.1 ± 0.5*	450.3 ± 45.4	1.8 ± 0.3	13.5 ± 4.5*	0.0412***
P150T1	115.5 ± 87.0***	9.3 ± 1.3***	22.8 ± 11.8***	3.3 ± 0.5	355.3 ± 166.0*	0.4 ± 0.2	8.7 ± 2.8	0.0435***
P150T3	543.6 ± 160.8	26.6 ± 3.1**	37.0 ± 12.4*	2.1 ± 1.1*	651.2 ± 150.7	1.5 ± 0.3	10.2 ± 0.5	0.0248***
P400T1	797.3 ± 193.3	83.0 ± 21.2	91.7 ± 32.9	16.8 ± 3.7	573.1 ± 133.3	0.8 ± 0.1	-0.2 ± 5.9	0.166*
P400T3	805.3 ± 216.2	59.7 ± 26.5	97.8 ± 16.6	1.5 ± 1.1**	792.0 ± 34.5	0.4 ± 1.0	-3.3 ± 3.17	0.0423***
P1000T1	1098.1 ± 184.1	51.1 ± 22.0	71.57 ± 18.8	4.8 ± 6.2	515.6 ± 353.5	0.6 ± 1.8	1.9 ± 7.4	0.4515
P1000T3	n.d.	n.d.	n.d.	n.d.	n.d.	n.d.	n.d.	0.3826

MBC–Microbial biomass Carbon, MBN–Microbial biomass Nitrogen

n.d.—not determined

*a* after log of percentage values

The only microbial parameter that increased significantly among the most contaminated sites inside the area was potential nitrification. Although being considered one of the most sensitive soil microbial processes regarding metal stress [70], some studies have shown adaptation of nitrifying populations at metal-contaminated sites [71], which may be the case in the present study. Nevertheless, high nitrification rates may indicate an unbalance in the N-cycling, which may result in losses of N from the system by leaching or denitrification. Recently disturbed ecological systems tend to show greater nitrification rates, which decreases along the successional status [72].

Although some authors (e.g., [73]) do not recommend the inclusion of microbial parameters in ssERA, because microbial communities demonstrate functional redundancy, rapid changes across small spatial scales, and high sensitivity for confounding factors (e.g., moisture, nutrients), the present study is in agreement with reports on decreases in microbial enzyme activity [74], carbon biomass and basal respiration [74–76] in impacted soils. Consequently, these parameters seem to be useful tools for assessing metal effects on microbial functions in heavily contaminated areas. On the other hand, soil microbial communities and the key biological processes they mediate are closely related to vegetation and soil use [77, 78]. The failure in the vegetation establishment inside the smelter area, especially in the tailing deposits, not only due to metal toxicity but also to inappropriate soil physical and chemical properties, may also contribute to the observed decreases in microbial activity and biomass. Given that key microbial processes on C, N and P cycling have most likely been impaired due to such conditions as well as to the high metal contents, the maintenance of vegetation in these heavily-contaminated sites will be progressively more difficult, leading to intensification of erosion processes and dispersion of pollutants [70].

### 3.7 Organic material decomposition (EcoLoE)

When evaluating litter breakdown, the validity criterion of 60% mass loss in the reference treatment at the end of the study [51] was fulfilled. As for the decomposition of the organic material, the monthly decay rate in the contaminated sites within the smelter area was significantly lower than in the overall reference (Table 5). Only sites located 1,000 m away from P0 presented higher monthly decay rates than the overall reference. Moreover, a significant negative correlation was also found between the monthly decay rate and metal contamination [47]. According to the threshold value proposed by Römbke et al. [51] of more than 25% difference in mass loss between reference and contaminated sites to signal the presence of significant effects, all sites within the smelter area did exceed this level; differences in mass loss relatively to the overall reference ranged between 31% and 64% after 131 d of exposure [47].

The present results corroborate the results reported in previous studies revealing significant effects of metal (e.g., [79]) or pesticide contamination [80] on the decay rate of organic material in soil, even though other studies showed transient or no effects under some stressors, (e.g., [49, 81, 82]).

Also, the reduced microbial activity, faunal feeding activity (data presented at Niemeyer et al. [29]) and density of detritivores, in combination with the low moisture and high temperatures at the more exposed sampling points (due to low vegetation cover), may have contributed to reduce litter decomposition within the smelter area [78]. Thus, the effects on litter decomposition observed in the present study may be attributed not only to a toxic effect caused by metal contamination on microbial and faunal communities, but also to indirect effects leading to non-suitable habitat conditions for soil fauna and microbial communities.

### 3.8 Risk values for each line of evidence and integrated risk

#### 3.8.1 Risk to retention function

The individual contribution from each parameter and the combined risk values from each LoE (ChemLoE and EcotoxLoE) for the soil retention function is shown in Table 6. Low risk values (between 0.25 and 0.50) were found for the ChemLoE, except at sampling points P1000T1 and P150T1, as anticipated from the extractable concentrations of a few metals above the groundwater intervention values proposed by VROM [55]. From the EcotoxLoE, reproduction test with *D*. *magna* pointed low and high (> 0.75) risk values for P150T1 and P1000T1, respectively, and no risk (< 0.25) was indicated for the other sites, which is in accordance to the low metal contents in the soil extracts from all except the latter two soils.

**Table 6:** Individual and combined risk values from the chemical (ChemLoE) and ecotoxicological (EcotoxLoE) lines of evidence and the integrated risk (IR) for the soil retention function.

	Chem LoE (Extractable metals)	Growth *Pseudokierchneriella subcapitata*	Reproduction *Daphnia*. *magna*	Combined EcotoxLoE	IR Retention Function
**Group 1**					
1000T1	0.99	0.16	1.00	0.91	0.96
20T3	0.00	0.00	0.00	0.00	0.00
400T3	0.00	0.00	0.00	0.00	0.00
**Group 2**					
P0	0.00	0.04	0.08	0.06	0.03
20T1	0.00	0.08	0.15	0.12	0.06
150T1	1.00	0.20	0.41	0.32	0.99
50T3	0.44	0.06	0.03	0.04	0.27
**Group 3**					
50T1	0.00	0.06	0.14	0.10	0.05
400T1	0.00	0.10	0.04	0.07	0.03
150T3	0.00	0.08	0.00	0.04	0.02
1000T3	0.00	0.04	0.00	0.02	0.01

As a result, a risk for the retention function of the soil was only found at sampling points P150T1 and P1000T1 which was previously demonstrated in tier 1 through the *D*. *magna* lethal test [29]. In the case of sampling point P1000T1, by being in the middle of a pasture area outside the smelter area, the presence of another type of contamination (e.g., fertilizers, pesticides) causing effects on aquatic organisms should not be ruled out (as also pointed out in some of the ecotoxicological data with soil organisms in section 3.2).

Low risk for the retention function indicates low mobility of metals from soil to water. This may be favored by the type of soil in the region, which is rich in expansive clay (montmorillonite) [34], with a probable high adsorption potential, accentuated by neutral pH values and ageing. Soil characteristics in this smelter area facilitate the capture of metals, especially in a wetland zone, not allowing their mobilization into groundwater [83]. Nevertheless, the low metal extractability was probably related to the metal form: as pointed out by Andrade Lima and Bernardez [84] when studying the leaching of the slag in the smelter area in Santo Amaro, Pb, Zn, Cd, and other potentially toxic elements, were relatively stable in a weak acidic environment for short contact times, most likely due to the low leachability of the metallic Pb and the Zn-bearing species. Thus, in some sites in neighboring areas where groundwater could be used for human consumption, groundwater monitoring would be advisable, especially at those sites where metal concentrations are very high and where the soils are more permeable.

#### 3.8.2 Risk to habitat function

Tables 7 and 8 show the individual contribution and the combined calculated risk values for each LoE in habitat function. Sampling points presenting very high habitat function risk values (above 0.75) or moderate risk values (between 0.50 and 0.75) were those where the total metal concentrations exceeded the HC50_cor_ values (P0, P150T1 and P50T3) or were near that threshold (P150T3) in the ChemLoE. Regarding the EcotoxLoE, the differences in sensitivity of the test species and endpoints were clearly visible. Reproduction with Oligochaeta species *E*. *andrei* and *E*. *crypticus* were the most sensitive tests. Both oligochaete species indicated high risk values in points P150T1 and P1000T1, and moderate risk in P50T3. However, the EcotoxLoE integrating these results with reproduction of *F*. *candida* and plant endpoints presented moderate ecotoxicological risk to P150T1 and P1000T1, while low risk values (≤0.50) to other points. The risk values to soils of group 2 could have been underestimated because the unexpected low reproduction rates of *F*. *candida* in Ref. 2. The highest risk value was found in P150T1, the most contaminated soil with a sandy texture (sampling point from group 2). In the case of sampling point P1000T1, these results also indicated, as those of the retention function in the present study, and avoidance tests with *E*. *andrei* in tier 1 [29], a possible presence of an unknown contamination.

**Table 7:** Individual and combined risk values from the chemical and ecotoxicological line of evidence (EcotoxLoE) for the soil habitat function.

	Chem LoE (total metals)	Reproduction *Folsomia candida*	Reproduction *Enchytraeus crypticus*	Reproductio *Eisenia andrei*	Shoot length *Avena sativa*	Shoot length *Brassica rapa*	Dry Weight *Avena sativa*	Dry Weight *Brassica rapa*	Combined Ecotox LoE
**Group 1**									** **
1000T1	0.00	0.03	0.96	0.93	0.00	0.17	0.00	0.58	0.63
20T3	0.23	0.38	0.00	0.23	0.42	0.15	0.32	0.59	0.32
400T3	0.05	0.43	0.00	0.00	0.28	0.28	0.08	0.63	0.28
									
**Group 2**									
P0	0.85	0.00	0.39	0.31	0.00	0.00	0.00	0.00	0.12
20T1	0.25	0.00	0.59	0.26	0.00	0.00	0.00	0.00	0.16
150T1	1.00	0.00	0.99	0.92	0.17	0.00	0.14	0.53	0.71
50T3	1.00	0.00	0.59	0.66	0.14	0.00	0.00	0.27	0.29
									
**Group 3**									
50T1	0.19	0.04	0.00	0.21	0.27	0.09	0.36	0.17	0.17
400T1	0.49	0.00	0.00	0.16	0.00	0.00	0.00	0.00	0.02
150T3	0.72	0.06	0.00	0.83	0.17	0.33	0.29	0.58	0.41
1000T3	0.06	0.61	0.00	0.30	0.11	0.00	0.00	0.21	0.21

**Table 8:** Individual and combined risk values from the ecological line of evidence (EcolLoE) for the soil habitat function.

Soil groups	Microbial parameters							Surface dwelling arthropods						
	MBC	MBN	Asparaginase	DHA	Ac Fosf	Amon	Nitrif	Araneae	Hymenoptera	Coleoptera	Orthoptera	Other Orders	Decomp	Combined Ecol LoE
**Group 1**														** **
1000T1	0.41	0.02	0.16	0.34	0.16	0.15	0.00	0.86	0.42	0.32	0.42	0.45	0.41	0.37
20T3	0.73	0.63	0.16	0.81	0.28	0.54	0.00	0.63	0.50	0.45	0.24	0.26	n.d.	0.46
400T3	0.20	0.16	0.13	0.80	0.22	0.39	0.00	0.65	0.12	0.26	0.29	0.60	0.84	0.44
**Group 2**														
P. Zero	0.72	0.89	0.37	0.90	0.56	0.60	0.74	0.76	0.41	0.27	0.25	0.76	0.82	0.69
20T1	0.61	0.74	0.81	0.82	0.68	0.34	0.69	0.86	0.57	0.74	0.18	0.29	n.d.	0.63
150T1	0.82	0.81	0.73	0.54	0.42	0.48	0.55	0.69	0.57	0.82	0.53	0.76	0.84	0.69
50T3	0.28	0.56	0.62	0.70	0.27	0.62	0.71	0.88	0.49	0.48	0.47	0.53	0.84	0.62
**Group 3**														
50T1	0.36	0.78	0.87	0.83	0.62	0.61	0.77	0.90	0.66	0.13	0.22	0.18	0.76	0.67
400T1	0.19	0.40	0.57	0.57	0.07	0.12	0.00	0.48	0.23	0.67	0.25	0.49	0.38	0.37
150T3	0.15	0.47	0.56	0.70	0.05	0.53	0.62	0.99	0.52	0.07	0.29	0.18	0.91	0.47
1000T3	n.d.	n.d.	n.d.	n.d.	n.d.	n.d.	n.d.	0.54	0.45	0.44	0.13	0.29	0.31	0.38

MBC–Microbial biomass Carbon.MBN–Microbial biomass nitrogen. DHA–Dehydrogenase. Ac Fosf—Acid phosphatase. Amon–Ammonification. Nitrif–Nitrification rate. Decomp–Decomposition of organic material

n.d.—not determined

Among the parameters from the EcoLoE, some microbial parameters, namely BMC, BMN, acid phosphatase, asparaginase, and nitrification rate were the most sensitive endpoints in discriminating contaminated sites (this statement was also based on previous analyses carried out in Niemeyer et al. [46, 47]. Bacterial growth/biomass was highly rated by Critto et al. [8] as parameters to be assessed in all Triad tiers, mainly due to their rapidity and low cost. Regarding soil surface dwelling invertebrates, high risk values (>0.75) were indicated only by Araneae in points P0, 20T1, 50T1, 50T3 and 1000T1, and Others (pooled data of other groups) in points P0 and P150T1. In general, these soil fauna parameters did not demonstrate the same level of sensitivity as the other ecological parameters. Abundance and morphospecies richness of main groups of surface running invertebrates were not sensitive parameters to discriminate metal contaminated sites. Similarly to our findings, abundance and number of taxa, as far as diversity indices (see [47]), were also not sensitive to contamination in Semenzin et al. [85]. This can be explained by the high mobility of surface dwelling organisms in comparison to soil dwelling invertebrates, not presenting a relation with properties of a particular site but rather with characteristics of a larger area around the sampling point. More elaborated conclusions could be taken, namely in terms of effects to particular functional groups and to find better cause-effect relationships, if identification would be done with soil dwelling organisms and with a higher taxonomic resolution (families, species). Besides being more detailed this information could also be used to apply a trait-based approach which could help to better understand possible effects on the functioning of the ecosystem [86].

**Fig 2:**
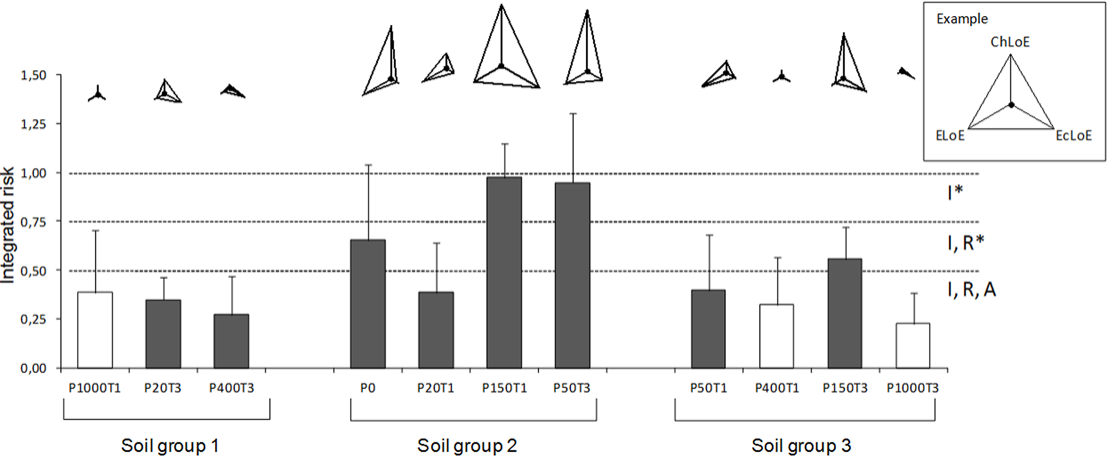
Integrated ecological risk values for habitat function (+ standard deviation) (Min,0; Max, 1) for each sampling point, combining information from the chemical (ChemLoE), ecotoxicological (EcotoxLoE), and ecological (EcoLoE) lines of evidence. Points with grey bars are located inside of the smelter area. Different bands indicate limits of accepted risk values for different soil uses (A agriculture, R residential, I industrial; asterisks indicate necessity of sealed soils) according to Jensen and Mesman (2006). Triangles on top of each bar represent the contribution of each LoE for the integrated risk value being an indicator of the weight of evidence (a triangle with equal sized arms (equilateral) indicates a similar risk value (high weight of evidence) for each LoE). The length of each “arm” of the triangle is proportional to the risk value for each LoE (on the top right the example with the length of each axis of the triangle representing maximum risk (1) from each LoE).

Integrated risk values (IR) are shown in Fig 2. The tier 2 confirmed the low risk (IR≤0.50) pointed by tier 1 [29] in sampling points P50T1, P400T1, P1000T1, P20T3, P400T3, and P1000T3, all clay based soils, where all the lines of evidence pointed into the same direction, except in P50T1, where the EcoLoE indicated a moderate risk.

In P150T3, tier 2 showed the same level of moderate risk (0.51≤IR≤0.75) indicated in tier 1, but with a lower standard deviation, which means that the three LoE are indicating the same level of risk. Lower standard deviation in habitat function of tier 2 was related to the risk calculation carried out separately for habitat and retention functions. As in P150T3, sampling point P20T1 showed a moderate risk (0.51≤IR≤0.75) in tier 1, also with a slightly higher level of uncertainty, while in tier 2 it was considered as a lower risk (IR 0.4), but the same level of uncertainty remained. This can be explained by the low risk indicated both by the ChemLoE and the EcotoxLoE in tier 2, which did not agree with the moderate risk pointed by EcoLoE. The higher risk values pointed in tier 1 at these points could be related to the type of tests used, especially the risk value obtained with the avoidance test with *E*.*andrei*.

The integrated risk for the habitat function in tier 2 confirmed the spatial heterogeneity of the study area, already demonstrated by the results of tier 1 [29]. In the same way, high levels of risk were found at sampling points within the smelter area, particularly in soils with a coarse texture (soils from group 2; Fig 2). Very high integrated risk values (IR>0.75) were calculated for sampling points P150T1 and P50T3, corresponding to tailing deposits. According to the Dutch contaminated-site policy [1, 87], these high risk values restrict the use of the area even to industrial activities. The standard deviation found in the final risk number for the habitat function in points P0 and P50T3 is related to the high risk levels indicated by chemical analysis, to the low toxicity indicated in plant endpoints and reproduction of *F*. *candida*, and the inability of some ecological parameters, namely those related to surface dwelling invertebrates, in discriminating ecological risk levels. These results confirm the added value of not only integrating information from different lines of evidence, but also in using different indicators inside each LoE. This will provide more detailed information about uncertainties in the ssERA, and demonstrate the inability of a purely chemical based assessment to reduce uncertainty and in predicting the integrated risk of a contaminated site (Rutgers and Jensen 2011). Moreover, these results reinforce the statement that information from the Triad can be used as a basis for decisions about remediation actions and management concerning the future of a site.

### 3.9 Uncertainty analysis

In this section the main sources of uncertainty regarding the performed risk assessment are identified and discussed. Uncertainty related to data quality can be discarded in case of ecotoxicological tests and ecological parameters. Only data from validated tests were used, and the adoption of standardized sampling protocols to sample soil and soil epigeic invertebrates, and to determine microbial parameters, guarantee the validity of ecological data. Regarding chemical data, the low detection limit on the extractable metals could have led to an underestimation of extractable metal concentrations and consequently to an underestimation of the risk in the chemical line of evidence for the soil “retention” function. However, the low toxicity observed in the vast majority of points allows to say that, even with underestimated values, the actual risk towards the soil retention function is not high. Nevertheless, a high level of detection would be desirable. Still, in the ChemLoE, the use the Dutch screening levels as screening values to calculate the Toxic Pressure could be a source of uncertainty per se. However, the total metal concentrations in soils were compared with the HC50cor (Dutch HC50EC50 values [52]), values that were corrected for sampling site-specific differences, taking into account the organic matter and the clay content of each soil, thus reducing the uncertainty. In Brazil, soil quality values present in the Federal legislation (that appeared after conducting this study) are based on the Dutch values but without any correction to soil properties, and were originally adapted to São Paulo state. Therefore, their straightforward application in this case could have brought even a higher level of uncertainty. This is a clear indication that, within Brazil, region-specific screening levels should be developed in the future.

Some uncertainty can be attributed to the use of standard species in ecotoxicity tests. It was assumed that these species are sensitive enough to protect the community present in the site. However, the very few studies comparing the sensitivity between e.g., the traditional soil invertebrate species used in ecotoxicological testing with autochthonous soil invertebrate species, show that the differences in sensitivity are not so high (e.g., [88]). Therefore, the use of standard test species (that also exist in Brazil), is still the best way to tackle the problem with an acceptable level of uncertainty. Regarding plant species, the use of crop species instead of wild species could be a source of uncertainty. However, the development of ecotoxicological tests with wild species is still in its beginnings, especially for chronic tests, meaning that testing crop species is still the best available approach for a routine based assessment, despite the level of uncertainty associated. In this case, testing more plant species would be desirable to increase the sensitivity range possibly covering wild species. Nevertheless, due to the high sensitivity of the two species tested, the bias could be towards an overestimation of effects.

Regarding the ecological parameters related to soil invertebrates, the fact that only epigeic organisms were sampled, could have led to an underestimation of effects towards in-soil organisms. This statement is based on expert knowledge of the ecology of these organisms and on the knowledge of the study area, which could indicate a more pronounced effects on in-soil invertebrates (generally more sensitive not only to habitat configuration but also soil properties and metal loadings) than those observed for epigeic invertebrates.

In the present work, equal weight was attributed to all parameters and to the three LoEs. However, aiming to reduce uncertainty in some risk values for some of the sampling points (e.g., P1000T1), a different weight could have been given to some parameters with a high level of uncertainty (in this example, the high effects observed in the ecotoxicological testing due to other stressors than metals). However, with the amount of information used for all three LoE (chemistry, toxicology and ecology), and by using a “weight of evidence” approach, we consider that uncertainty is minimized and the differences between the outcome of the individual LoE should hence be reduced.

One last aspect dealing with uncertainty is related to the sampling design adopted in this study and the possibility to extrapolate the conclusions drawn for the entire area. Spatial extrapolation was not envisaged in this study since it would bring some level of uncertainty. However, due to the extensive knowledge of the area, we believe that the conclusions taken from the data obtained can be extrapolated with some security to other places within the area having similar habitat configuration and metal loadings. Nevertheless, to help risk managers in this context, a spatial assessment of the risk, using the information gathered in this study, is ongoing.

## Conclusions

In general, results in tier 2 of the ssERA confirmed the high (probably environmentally unacceptable) risk levels in the smelter area already indicated in tier 1, and associated with tailing deposits, but with a further reduction of uncertainties. Locations outside the smelter area demonstrate lower or acceptable risks. In the same way as in tier 1, the low toxicity in eluate tests indicated high adsorption of metals in soil, probably favored by content and type of clay, ageing and neutral pH, and consequently negligible risk due to the high retention capacity in most sampling points. Results of chemical analysis of extracts confirmed the low mobility of metals from soil to water. Moreover, the present results indicate that the current cover of the tailing deposits failed to restore the site by not creating appropriate conditions for the establishment of plant (revegetating) and microbial and animal communities inside the area. So, besides the direct effects of metal contamination, also indirect effects are visible from the presence of these contaminants, compromising the functioning of the ecosystem inside the smelter area. High risk values (IR > 0.75) in habitat function inside the smelter area indicate the need to proceed with some remediation action, such as an improved encapsulation of tailing deposits and recovery of the vegetation. These actions could not only improve soil conditions and ecosystem functioning, but they could mainly avoid the transport of contaminants to other environmental compartments, namely via dust dispersal to outside the area, or via surface runoff to the existing temporary ponds and the Subaé river.

## Supporting Information

S1 File Detailed explanation and formulas used for risk calculations. (DOCX)
