# Supplementary material for: Ecological Risk Assessment of a Metal-Contaminated Area in the Tropics. Tier II: Detailed Assessment
Source: PLoS One. 2015 Nov 3;10(11):e0141772. doi: 10.1371/journal.pone.0141772 (PMC4631348; doi:10.1371/journal.pone.0141772)
Supplement: S1 File — (DOCX) [file pone.0141772.s001.docx]

**S1 File**

**Ecological Risk Assessment of a Metal-Contaminated Area in the Tropics. Tier II: Detailed Assessment.**

**Detailed explanation and formulas used for risk calculations**

Risk calculations adopted in this study are based on the approach proposed by Jensen and Mesman [1]. As explained in section 2.11.3, risk values were calculated for each sampling point and for each parameter assessed at each LoE. These values were expressed in a scale between 0 (“no risk”) and 1 (“highest risk”), and they were normalized for the risk value of the corresponding reference point (assumed to be zero). It implies that, before risk calculations, all results from the different parameters should be made comparable (expressed on the same scale) across the various LoE. The different steps and formulas used in risk calculations are detailed below.

1. **Chemical Line of Evidence (ChemLoE)**

**1.1. ChemLoE for the soil “habitat” function**

The first step was to calculate the Toxic Pressure (expressed as the Potential Affected Fraction of species - PAF) at each sampling point using the approach from Rutgers et al. [52]. It was assumed that each metal had an independent toxic mode of action, therefore the Toxic Pressure (TP) for each metal *i* at each sampling point *j* (M_ij_) was calculated using the following formulas:

${TP}_{Mij}=\frac{1}{1+e^{-\log\left( {HU}_{ij} \right)\times\beta_{i}^{-1}}}$ (eq. 1)

${HU}_{ij}=\frac{[n_{ij}]}{{10}^{\alpha i}}$ (eq. 2)

where HU*_ij_* is the hazard units for M*_ij_*; [n*_ij_*] is the concentration of M*_i_* in sampling point *j* in mg/kg; α*_i_* is the log transformed toxicity benchmark for M*_i_* (in this case Log HC50 after correction for soil organic matter and clay contents [52]); β*_i_* is a model parameter for the SSD for M*_i_* toxicity data [52].

Afterwards, the TP for the mixture of metals at each sampling site *j* (TP*_MMj_*) was calculated using the following formula and assuming a response addition pattern [52]:

${TP}_{MMj}=1-(1-{TP}_{M1j})\times(1-{TP}_{M2j})\cdots(1-{TP}_{Mnj})$ (eq. 3)

where TP*_Mij_* is calculated from equation 1.

The next step was to scale the TP*_MMj_* value for each sampling point within the group of sampling points in relation to the corresponding reference point (TP*_MMRef_* is set to 0), according to the following formula [1]:

${Risk}_{ChemLoE(j)}=({TP}_{MMj}-{TP}_{MMRef})/(1-{TP}_{MMRef})$ (eq. 4)

The calculated Risk*_ChemLoE(j)_* value is considered the risk value for the ChemLoE for sampling point *j* and that was used to calculate the integrated risk (see below).

**1.2. ChemLoE for the soil “retention” function (ChemLoE(ext))**

For the ChemLoE(ext) of the retention function, results from each extractable metal *i* at each sampling point *j* (n*_ij_*) were compared to water quality objectives (α*_i_*) from VROM [55] to obtain as risk ratio (Risk*_ij_*). This was then scaled against the metal ratio value from the respective reference soil (Risk*_iRef_*) to obtained the scaled risk value (Risk*_ij(s)_*) that was then integrated into the overall risk for the ChemLoE(ext) for extractable metals for each point (Risk*_ChemLoE(i)ext_*). The following formulas were used [1]:

$Rij=\frac{[n_{ij}]}{\alpha_{i}}$ (eq. 5)

${Risk}_{ij}=1-(1/(1+R_{ij})$ (eq. 6)

${Risk}_{ij(s)}=({Risk}_{ij}-{Risk}_{iRef})/(1-{Risk}_{iRef})$ (eq. 7)

${Risk}_{ChemLoE(j)ext}=1-(1-{Risk}_{1j(s)})\times(1-{Risk}_{2j(s)})\cdots(1-{Risk}_{nj(s)})$ (eq. 8)

1. **Ecotoxicological Line of Evidence (EcotoxLoE)**

**2.1. EcotoxLoE for the soil “habitat” function**

Results of the ecotoxicological tests with soil invertebrates and plants were used (absolute data on reproduction of *E. andrei*, *E. crypticus* and *F. candida*, and of *A. sativa* and *B. rapa* growth, both as shoot length and biomass). In this case it is expected a negative response in relation to the one from the corresponding reference soil. Each value obtained for test *i* on site *j* (Test*_ij_*) was transformed (R*_ij_*) and then scaled (R*_ij(s)_*) in relation to the R value of the corresponding reference site (R*_iRef_*). These scaled values were them used to calculate the risk value for the EcotoxLoE for each sampling point *j* (Risk*_EcotoxLoE(j)_*). The following formulas were used [1]:

$Rij=\frac{{Test}_{ij}}{100}$ (eq. 9)

$R_{ij(s)}=(R_{ij}-R_{iRef})/(1-R_{iRef})$ (eq. 10)

${Risk}_{EcotoxLoE(j)}=1-({10}^{\frac{\sum_{i}^{n} Log \left( 1-R_{ij\left( s \right)} \right)}{n}})$ (eq. 11)

**2.2. EcotoxLoE for the soil “retention” function**

Results of the ecotoxicological tests with eluates on *D. magna* reproduction and *P. subcapitata* growth, expressed as the percentage of inhibition in comparison to the control, were used. In this case it is expected a positive response in relation to the one from the eluate of the corresponding reference soil. Each value obtained for test *i* on site *j* (Test*_ij_*) was transformed (R*_ij_*) and then scaled (R*_ij(s)_*) in relation to the R value of the corresponding reference site (R*_iRef_*). These scaled values were them used to calculate the risk value for the EcotoxLoE(ext) for each sampling point *j* (Risk*_EcotoxLoE(j)ext_*). The following formulas were used [1]:

$Rij=\frac{{100- Test}_{ij}}{100}$ (eq. 12)

$R_{ij(s)}=(R_{ij}-R_{iRef})/(1-R_{iRef})$ (eq. 13)

${Risk}_{EcotoxLoE(j)ext}=1-({10}^{\frac{\sum_{i}^{n} Log \left( 1-R_{ij\left( s \right)} \right)}{n}})$ (eq. 14)

1. **Ecological Line of Evidence (EcoLoE)**

**3.1. Risk values for microbial parameters and organic matter decomposition**

Individual results from the microbial parameters and organic matter decomposition were used. In this case it is expected a negative response in relation to the one from the corresponding reference soil. Each value obtained for parameter *i* on site *j* (Par*_ij_*) was transformed (R*_ij_*) and then scaled (R*_ij(s)_*) in relation to the R value of the corresponding reference site (R*_iRef_*). These scaled values were them used to calculate the risk value for the EcoLoE for each sampling point *j* (see below). The following formulas were used [1]:

$Rij=\frac{{Par}_{ij}}{100}$ (eq. 15)

$R_{ij(s)}=(R_{ij}-R_{iRef})/(1-R_{iRef})$ (eq. 16)

**3.2. Risk values for soil invertebrates**

Data on abundance and morphospecies richness of the most frequently soil surface dwelling groups (Araneae, Hymenoptera, Coleoptera, Orthoptera) were used separately, while data on other groups, including Isopoda, Dermaptera, Hemiptera, Diplopoda and Mantodea, were pooled. Since both abundance and number of morphospecies are the result of the same survey, the BKX_Triad method [1] was used. This method allows integrating information from different ecological observations into a single risk value, even if the original data has different units. So each value obtained for abundance and richness of group *i* on site *j* (Par*_ij_*) was scaled to the reference values (Par*_iRef_*) to obtain a ratio value (R*_ij_*). These were then used in the BKX_Triad formula (BKX*_ij_*). These were then used to in combination with the microbial and decomposition data obtained from Eq. 16 to calculate the risk value for the EcoLoE for each sampling point *j* (see below). The following formulas were used [1]:

$Rij=\frac{{Par}_{ij}}{{Par}_{iRef}}$ (eq. 17)

${BKX}_{ij}=1-({10}^{\frac{-\sum_{i}^{n} Log \left( R_{ij} \right)}{n}})$ (eq. 18)

**3.3. Deriving risk values for the EcotoxLoE**

R*_ij(s)_* (Eq. 16) and BKX*_ij_* (Eq. 18) values for each ecological parameter at each sampling site were then integrated to obtain the risk value for the EcoLoE for each sampling point *j* (Risk*_EcoLoE(j)_*). The following formula was used [1]:

${Risk}_{EcoLoE(j)}=1-({10}^{\frac{\sum_{i}^{n} Log \left( 1-R_{ij\left( s \right)} OR {BKX}_{ij} \right)}{n}})$ (eq. 19)

1. **Integrated risk (IR)**

The integrated risk for each sampling point (IR*_j_*) was calculated using the risk values obtained from each line of evidence (Risk*_ChemLoE(j)_*, Risk*_EcotoxLoE(j)_*, and Risk*_EcoLoE(j)_*). The same process was performed for the soil “retention” function, by integrating Risk*_ChemLoE(j)ext_*, Risk*_EcotoxLoE(j)ext_* values. The following formula was used [1]:

${IR}_{j}=1-({10}^{\frac{Log \left( 1-{Risk}_{ChemLoE\left( j \right)} \right)+Log \left( 1-{Risk}_{EcotoxLoE\left( j \right)} \right)+Log \left( 1-{Risk}_{EcoLoE\left( j \right)} \right)}{3}})$ (eq. 20)

To evaluate whether the different LoEs contributed differently to the total risk, the standard deviation associated to each IR value was also calculated.
